# UPLC-ESI-MS/MS Based Characterization of Active Flavonoids from *Apocynum* spp. and Anti-Bacteria Assay

**DOI:** 10.3390/antiox10121901

**Published:** 2021-11-27

**Authors:** Gang Gao, Ning Liu, Chunming Yu, Ping Chen, Jikang Chen, Kunmei Chen, Xiaofei Wang, Bin Liu, Aiguo Zhu

**Affiliations:** 1 Institute of Bast Fiber Crops, Chinese Academy of Agricultural Sciences, Changsha 410205, China; gaogang@caas.cn (G.G.); liu7ning2628@163.com (N.L.); yuchunming@caas.cn (C.Y.); chenping02@caas.cn (P.C.); chenjikang@caas.cn (J.C.); chenkunmei@caas.cn (K.C.); wangxiaofei@caas.cn (X.W.); 2 College of Biology, Hunan University, Changsha 410082, China

**Keywords:** *Apocynum* spp., flavonoids, antioxidant, antimicrobial, wound healing

## Abstract

In the current study, the active flavonoids from *Apocynum venetum* and *Apocynum hendersonii* leaf were efficiently characterized using UPLC-ESI-MS/MS, and yielding the highest content of 15.35 mg/g (*A. venetum)* and 13.28 mg/g (*A. hendersonii*) respectively. The antioxidant assay in vitro showed that the isolated flavonoid ingredient groups exhibited free radical scavenging activities to DPPH, ABTS and linoleic acid. The antimicrobial assay revealed the isolated flavonoid ingredient from both *A. venetum* and *A. hendorsonii* have exerted anti-MRSA and anti-*P. aeruginosa* effect through disrupting cell integrity and declining ATP. In vivo assay demonstrated that these flavonoid ingredients effectively accelerated MRSA-infected and *P. aeruginosa*-infected Balb/c mice wound healing. In summary, these results showed that the characterized flavonoid ingredients exhibited great potential as natural antioxidant and antimicrobial agents, and shed light into future potential applications of *Apocynum* spp.

## 1. Introduction

*Apocynum* spp., commonly known as “Luobuma” in Chinese is a perennial herbaceous widely distributed in saline–alkali land, riverbanks, sandy soils and fluvial plains [1]. As a genus of the plant family Apocynaceae [2,3], it is mainly divided into *Apocynum venetum* L. and *Apocynum hendersonii Hook.f* [4], two species with important medicinal perennial rhizomatous plants with ecological and economic value [2,5].

The utilization of *Apocynum* spp. was mainly focused on the textile application of phloem fibers [6], while chemical degumming technology for *Apocynum* spp. fiber preparation often caused environment pollution due to the use of chunk of chemicals [5]. Recently, mounting evidence has showed that the active components of *Apocynum* spp. have antioxidant [7], antibacterial [8], anti-fungal [9], anti-inflammatory [10] and sedation functions [11]. Moreover, the flavonoids extracted from plants can be used as effective and promising agents for inhibiting fungal infection [12]. Phytochemical investigation revealed that the biological activities of *Apocynum* spp. are closely related to its abundant flavonoids [13]. As a group of polyphenolic compounds consisting of a common C6-C3-C6 structure with two benzene rings, flavonoids can be subdivided into six major groups, including flavanols, flavanones, flavones, isoflavones, flavonols and anthocyanidins [10]. Compared with the pure natural compounds, the active flavonoids possess the advantage of playing its overall effect, such as simultaneously exerting comprehensive effects on the numerous biological targets [14].

How to efficiently obtain bioactive components with high efficiency, low toxicity, no resistance and biodegradation ability is still a challenge as the yields of these components were affected by various processing parameters. Meanwhile, the overall composition of the extract varied with the extraction protocols, plant species, organs (root, stem, leaves, root) and so on [15,16]. In addition, the use of extraction solvents, method of sample preparation, extraction duration and ratio of sample to extraction solvent also affected the composition and yielding of active compounds. Currently, the high-performance liquid chromatography (HPLC) is commonly used for the determination of effective components. Nevertheless, the limitations of low instrument resolution, poor sensitivity, or weak ultraviolet absorption of certain components have led to difficulties in the simultaneous detection of multiple components [17]. Hence, the simple, rapid and sensitive ultra-performance liquid chromatography electrospray ionization mass spectrometry (UPLC-ESI-MS/MS) method was developed and validated for the simultaneous determination of flavonoids or other metabolites [18,19].

In this work, the precise and active flavonoid ingredient rather than the total flavonoids form *A. venetum* (hyperoside, isoquercetin, quercetin-3-*O*-(6-*O*-malonyl)-galactoside, quercetin-3-*O*-(6-*O*-malonyl)-glucoside, and quercetin-3-*O*-(6-*O*-acetyl)-galactoside) and *A. hendersonii* (quercetin-3-sophoroside, isoquercetin, quercetin-3-*O*-(6-*O*-malonyl)-Galactoside) were efficiently characterized using the UPLC-ESI-MS/MS method. Their high antioxidant and antibacterial activities could shed light into future potential industrial applications using the two fiber and medicinal dual plants.

## 2. Materials and Methods

### 2.1. Plant Material

*A. venetum* and *A. hendersonii* were provided by the Institute of Bast Fiber Crops, Chinese Academy of Agricultural Sciences (Changsha, China) and the Western Agricultural Research Center, Chinese Academy of Agricultural Sciences, respectively.

### 2.2. UPLC-ESI-MS/MS Analysis and Isolation of Flavonoids

Accurately weighed sample (dry leaf powder, 5.0 g) was sonicated with 100 mL of 70% (*v*/*v*) ethanol for 30 min. Then, the crude extracts were centrifuged at 3000× *g* for 5 min. Finally, the supernatants were filtered through a 0.45 µm PTFE filter before UPLC-ESI-MS/MS analysis. HPLC grade solvents were purchased from Sigma Aldrich (St Louis, MO, USA). The 0.45 μm nylon membrane filter was purchased from Thermo Scientific (Schwerte, Germany).

The extracts were separated by the Waters UPLC HSS T3 phase column (100 × 2.1 mm, 1.7 μm). The binary mobile phase consisted of 0.1% aqueous formic acid (A) and acetonitrile (B). Internal standards (12 ng/mL, L-2-chlorophenylalanine) were added into 1 mL of extracts. The mixtures were vortexed for 30 s and transferred into ultrasonic cell disruption apparatus for 2 min, and then centrifuged at 13,000 rpm for 5 min at 4 °C. Next, 100 μL supernatant was transferred to brown LC injection bottle for analysis. The mass spectrogram was acquired by scanning the mass from C to D in the positive (+) and negative (−) ion modes using a MS system equipped with an ESI source. The positive (+) ion mode were as follows: CUR: 35; EP: 10; IS: 5500; CXP: 10; TEM: 600 °C; Gas1: 60; Gas2: 50. The negative (−) ion mode were as follows: CUR: 35; EP: −10; IS: −4500; CXP: −20; TEM: 600 °C; Gas1: 60; Gas2: 50. The integral peak area of metabolites was calculated according to the standard curve linear equation, and the absolute content of metabolites in real samples was obtained using the following equation [20].

The sample metabolite content (μg/g) = C × V × 10 − 3/M × N
(1)

where C represents the concentration value calculated by bringing the peak area of metabolites into the standard curve in the sample (μg/mL). The V represents constant volume (0.2 mL). The M and N stands for sample quality (g) and dilution multiple (5 times) respectively.

Both the *A. venetum* and *A. hendersonii* samples were separately solubilized in co-solvent as stock solutions for spectrophotometric assay. The spectra (200–450 nm) of the analytes (the isolated flavonoid ingredients) were obtained using a UV2401PC UV/visible spectrophotometer (SHIMADZU, Kyoto, Japan). Infrared spectra were detected within the wavelength range between 4000 and 400 cm^−1^ using a FTIR Nicolet iS50 FT-IR (Thermofisher, Waltham, MA, USA). Two samples and the standard sample of rutin were mixed with potassium bromide and pressed into transparent discs for FT-IR analysis. After the identification and quantity determination of flavonoids by UPLC-ESI-MS/MS analysis, the sample and solvent were increased proportionally (about 100 g air dried leaf were milled and extracted with 10 L of 70% ethanol at room temperature) for the large amount isolation of total flavonoids.

### 2.3. Antioxidant Assays

The free radical scavenging abilities of the samples were measured in three test systems using the stable radical ABTS^+^ [2,2-azino-bis-(3-ehylbenzothiazoline-6-sulfonic acid)], DPPH (2,2′-diphenyl-1-picrylhydrazyl) and inoleic acid/β-carotene bleaching assays. The inhibition percentage of ABTS^+^ (I*_ABTS+_*%), DPPH (I*_DPPH_*%) and bleaching (I*_bleaching_*%) were calculated according to the previously reported equations [21,22,23].

### 2.4. In Vitro Antimicrobial Assay

#### 2.4.1. Microbial Growth Conditions and Sample Preparation

Single colony of Methicillin-resistant Staphylococcus aureus, Gram-negative bacteria of *Pseudomonas aeruginosa* and fungi-*Aspergillus flavus* separately grew on the Luria Bertani (LB) agar plate (1% tryptone, 0.5% yeast extract, 1% NaCl, and 1.5% Agar, pH 7) was collected and dispersed in 5 mL fresh LB broth and incubated at 37 °C with 200 rpm rotation for 12 h. *A. flavus* was cultured at 30 °C in fresh Potato Dextrose Broth (3.5% PDB, Solarbio, Beijing, China) or PDA solid medium (4.6% PDA, Solarbio) with other conditions similar to that of bacteria. The cultured microbes were then used for the antimicrobial tests. All samples of the isolated flavonoids were filtered using sterile 0.22 μm filter membranes and all antimicrobial processes were completed on clean bench.

#### 2.4.2. Microbial Growth Conditions and Sample Preparation Inhibition Zone Test

Filter paper method was used to examine the antimicrobial activity against the three microbial strains. A total 100 µL of MRSA and *P. aeruginosa* suspension cultures (1 × 10^7^ CFU/mL) in the log phase were spread onto LB agar plates and *A. flavus* suspension (1 × 10^7^ CFU/mL) were swabbed uniformly onto Potato Dextrose Agar (PDA) agar plates using sterile cotton swabs. Filter papers (6 mm) immersed with 20 µL *A. venetum*, *A. hendersonii* or control group (cosolvent, dimethyl Sulfoxide) were transferred uniformly to the MRSA, *P. aeruginosa* and *A. flavus* agar plates. Thereafter, the agar plates were incubated at 37 °C overnight. After incubation for 12 h, the visible transparent halos on the plates were considered as the inhibition zones. The diameters and areas of the microbe-static ring were measured with Vernier caliper. Each sample was carried out in triplicates.

#### 2.4.3. Reduction of Cell Growth Test

Microbial (10^7^ CFU/mL) were inoculated with the isolated flavonoid ingredients of *A. venetum* and *A. hendersonii* with final concentration of 5.0 mg/mL in 1 mL centrifuge tube overnight. 100 µL of the three microbial suspensions were sucked and measured by a microplate reader every hour from 0 to 6 h. The microbial suspensions recorded were used to calculate the survival rate for the long-term bactericidal activity test. The tests were repeated three times for each sample extracts.

#### 2.4.4. Live/Dead Staining

The bacteria cells viability was investigated using double fluorescent dye method [24] with modification. The cells were inoculated with the isolated flavonoid ingredients of *A. venetum* and *A. hendersonii* in a total volume of 1.0 mL with final concentration of 5.0 mg/mL and maintained at 37 °C with 200 rpm shaking. Cosolvent was used as the control group. After treatment for 12 h, these cells were collected by centrifuging at 6000 rpm for 5 min and then re-suspended in fresh LB medium. Then, the two bacteria cells were co-stained with 1.0 mg/mL green fluorescent dye (Calcein-AM) for 5 min and then red fluorescent dye (PI) for 15 min in the dark state. These samples with different treatments were imaged under a Laser Scanning Confocal Microscope (FV1200, Olympus, Japan).

#### 2.4.5. Microbial Morphological Characterization

The MRSA, *P. aeruginosa* cells suspensions with a final concentration (10^7^ CFU/mL) were incubated with the isolated flavonoid ingredients of *A. venetum* and *A. hendersonii* for 12 h at 37 °C or 30 °C at 200 rpm. Cosolvent was served as control. After 12 h, these cells with different treatments were collected by centrifuging at 8000 rpm for 5 min and washed 2–3 times with PBS (PH 7.4), followed by fixing with 500 μL of 2.5% glutaraldehyde and then placed at 4 °C in dark for 4 h. The samples were thereafter washed three times with PBS and subsequently dehydrated once with different concentration of ethyl alcohol solutions of 30%, 50%, 70%, 80%, 90%, 95% and 100% for 10 min. In the end, the fixed samples were treated with gold sputtercoated and imaged with a scanning electron microscope (SEM-Hitachi S-3000N, Tokyo, Japan).

The metabolic activity was also evaluated using an Enhanced ATP Assay Kit (Beyotime). Briefly, 10^7^ CFU/mL of microbial cells were incubated with the isolated flavonoid ingredients of *A. venetum* and *A. hendersonii*. After different treatments, the cells were collected by centrifugation with a speed of 8000 rpm for 5 min at 4 °C and resuspended in the 100 µL of lysis buffer. The samples were transferred to an ultrasonic cell disruption apparatus for 5 min in an ice bath at 30% power with 3 s on/5 s off. Then, the collected upper liquid was monitored on a multi-mode detection platform under the luminance mode.

### 2.5. In Vivo Antimicrobial Assay

#### 2.5.1. Cytotoxicity and Biosafety Analysis

The cell cytotoxicity of the isolated flavonoid ingredients from the two species was assessed by MTT assay [25]. The proliferation test cells was based on color reaction of mitochondrial dehydrogenase in living cells. A mouse embryonic fibroblast cell lines (NIH-3T3) were purchased from the Xiangya Central Laboratory, Central-South University. Cells were grown in 75 cm^2^ culture bottles supplied with 5 mL DMEM (10% bovine serum, 1% penicillin, and 1% streptomycin) at a 5% CO_2_ at 37 °C and after a few passages, cells were seeded in 96-well plate (1 × 10^4^ cells per well). Different final concentrations of 5 and 10 mg/mL) of the total flavonoid from each of *A. venetum* and *A. hendersonii* were added in triplicate when the cells were in the logarithmic growth phase as experimental group. Cosolvent was served as the positive control group and untreated cells as a negative control. After incubation for 24 h, both the experimental and control groups were washed thrice with PBS to remove the dead cells and then added 100 μL of MTT solution (0.5 mg/mL) into each well. After 4 h incubation, 100 μL of DMSO were added, spun for 10 min at 37 °C to dissolve the crystal violet. Cell viability was then determined by measuring the optical density at 490 nm on the Microplate Reader in absorbance mode. The result was subjected to analysis using Origin 2018 (ANOVA).

#### 2.5.2. Hemolysis Assay

Hemolysis assay [26] was carried out on fresh Blab/c mouse blood samples. Pure fresh mouse blood cells were prepared by centrifuging at 3000 rpm, 4 °C for 5 min and washed two times using PBS (pH 7.4). A total 25 μL of red cells were then mixed with 475 μL of different concentrations of the isolated flavonoid ingredients of each of the two species, incubated for 4 h at 37 °C. Co-solvent was served as the negative control group and saline solution was used as a positive control. After centrifugation at 3000 rpm for 5 min at 4 °C, the red blood cell samples were taken out and photographed to record the results. The percentage of hemolysis of the supernatant was measured by using microplate reader with the absorbance set at 540 nm and calculated according to the following equation:

Hemolysis (%) = (Asample − Anegative)/(Apositive − Anegative) × 100%
(2)

where Asample means the absorbance values of supernatant of samples following the addition of the total flavonoid, Anegative is the absorbance values of cosolvent and Apositive is the absorbance values resulting from the addition of saline Solution.

#### 2.5.3. Microbial Infection Study

Animal Wound infection Model and Histological Analysis: All animal use procedures were authorized by Animal Ethical Committee of Hunan university Laboratory Animal Research Center (Changsha) according to the Guidelines outlined in the Declaration of Helsinki for all animal experimental investigations (Approval No. AEWC-20201029). The performance of the isolated flavonoids as wound dressings on infected wound of Blab/c mice was evaluated. Normal 7 weeks old female Balb/c mice with an average weight of 20 g were purchased from Hunan SJA Laboratory Animal Co., Ltd. (Changsha, China). After shaving and cleansing with 75% alcohol, a total of eighteen mice grouped into six groups (3 per group) were anaesthetized and circular wounds with a diameter of 13~15 mm to the panniculus carnosus were made on the backbones of the mice.

MRSA and *P. aeruginosa* were used to infect the wound. A total 100 μL of each of the two bacterial strains suspension (10^8^ CFU/mL) were separately smeared on the wounds surface of 9 randomly selected mice for each. The infection process was repeated for two more days, and then 100 μL of the isolated flavonoids were placed on the surface of the wound, each of the extracts to a group of three mice from each of the two infection group and fixed by medical bandaid (Yun Nan Bai Yao, Kunming, China). Cosolvent was served as the control to the three remaining mice from each of the two infected groups. Mice were imaged immediately after infection and treatment. The treatment procedure was repeated continuously for the next 14 days. Body weight of the mice and wound size were continuously monitored and recorded using electronic scale and Vernier, respectively, as well as photographed to evaluate the healing efficacy. The following formulation was used to calculate the wound area: width × length × π/4. Finally, all mice were killed and the tissues of infected wound areas and major organs (heart, liver, spleen, lung, kidney) were harvested for H&E stain pathology study [27].

#### 2.5.4. Histological Analysis and Blood Assay

The wound tissues and the five collected organs were each cut into 1 cm size, 4% paraformaldehyde solution added and kept for 24 h before being desiccated using ethanol and embedded in paraffin [28]. The tissues were further chopped into pieces about 5 μm and subjected for HE (hematoxylin-eosin) staining analysis. Histological images were taken by an inverted microscope (IX71, Olympus, Japan). Simultaneously, blood samples collected from all treatment group and control were centrifuged at 3000 rpm, 4 °C for 5 min and the supernatant was used for biochemical analyses.

### 2.6. Statistical Analysis

The data in the form of mean ± standard deviation for each treatment group were analyzed using one-way analysis of variance (ANOVA). Differences between groups with * *p* < 0.05, ** *p* < 0.01, and *** *p* < 0.001 were regarded as statistically significant. Image J and FV10-ASW4.2 viewer was used to analyze images. Vernier calipers were used to measure length and width. Origin 2018 software was used to plot graphs.

## 3. Results and Discussions

### 3.1. Characterization and Isolation of Total Flavonoids from A. venetum and A. hendersonii Leaf

**Figure 1:**
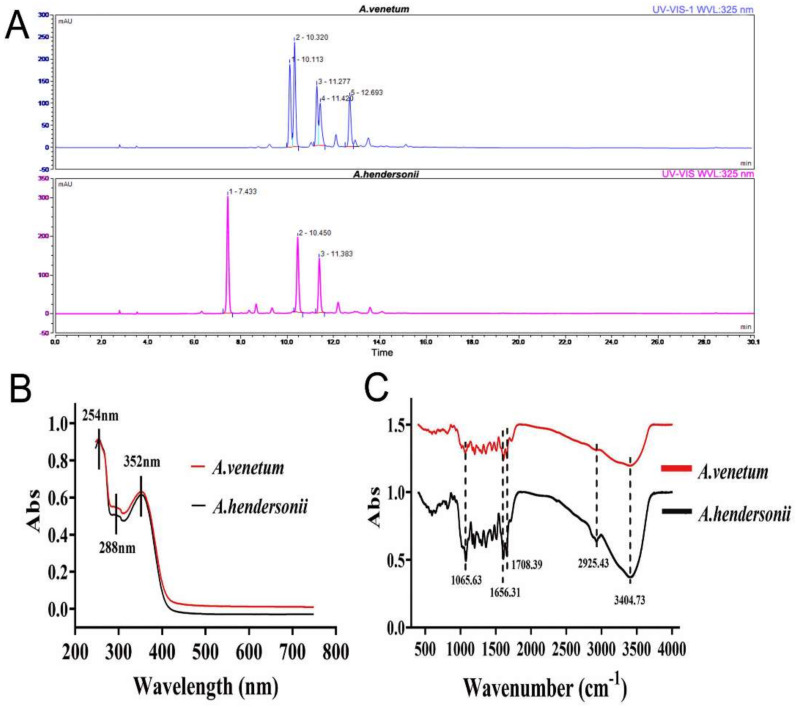
HPLC/UV chromatograms (**A**), UV-vis absorption spectra (**B**) and the FT-IR spectra (**C**) of the major tentative flavonoids in the extracts of *A. venetum* and *A. hendorsonii* leaf samples.

The components of *A. venetum* and *A. hendersonii* leaf samples were extracted by HPLC, and the presence of tentative flavonoids were identified by comparing the retention time and the corresponding peak of UV spectra with those of standards. The ultraviolet absorption spectra of flavonoid of *A. venetum* and *A. hendersonii* extracts with identical characteristic absorption peaks in the spectral range of 250–750 nm are shown in Figure 1A. The spectrum showed a very intense absorption in the 250 and 400 nm regions. It is inferred that the two tested constituents contain aromatic structure, which may be benzene ring(s). The obtained UV spectra (Figure 1B) of two samples were in a relatively good agreement with previous literatures [28,29]. The FT-IR spectra of the two samples shown in Figure 1C indicated the characteristic peaks corresponding to O-H stretch at 3404.73 cm^−1^, C-H stretch at 2925.43 cm^−1^, C=O of ester stretch at 1708.39 cm^−1^, aromatic ring stretch at 1656.31 cm^−1^ and C-O-C symmetric stretch at 1065.63 cm^−1^ All these characteristic peaks corresponded to the functional groups of flavonoids.

In the *A. venetum* samples, 5 main peaks were detected and showed maximum absorption in the UV spectra. While 3 main peaks were detected in the *A. hendersonii* samples (Figure 1A). To identify the chemical constituents of *A. venetum* and *A. hendersonii* samples, these tentative flavonoid components were collected for the ESI-MS/MS analysis. UV and MS data and the structural formula of the major flavonoid compounds obtained from the chromatograms of the two samples were summarized as Figure S1. Briefly, five and three major flavonoids were characterized from the ethanol extracts of *A. venetum* (hyperoside, isoquercetin, quercetin-3-*O*-(6-*O*-malonyl)-galactoside, quercetin-3-*O*-(6-*O*-malonyl)-glucoside, and quercetin-3-*O*-(6-*O*-acetyl)-galactoside) and *A. hendersonii* (quercetin 3-sophoroside, isoquercetin, quercetin-3-*O*-(6-*O*-malonyl)-galactoside) respectively, and tentatively identified by comparing their mass spectra with those of isolated flavonoids reported in previous literatures [29,30].

The content of these isolated flavonoid ingredients in the two sample were calculated, and reached 15.35 mg/g and 13.28 mg/g (dry weight) in *A. venetum* and *A. hendersonii* samples respectively (Table 1) After the previous characterization and quantity determination by UPLC-ESI-MS/MS analysis, large amount isolation and collection of the identified flavonoid ingredients were conducted by increasing the sample and solvent proportionally. All the isolated flavonoid ingredients exhibited potent concentration dependent activity to ABST^+^, DPPH and linoleic acid, and the IC_50_ values (the concentration with scavenging activity of 50%) were indicated in Table 1 (*A. venetum*: 201.21 ± 12.44, 35.68 ± 2.53, 31.02 ± 1.54; *A. hendersonii*: 195.47 ± 10.25, 33.52 ± 3.12, 24.15 ± 1.67). Although the isolated flavonoids showed moderate scavenging activity compared with the reported flavonoids from other plants [31], the activities were much higher than that of the positive control (rutin).

### 3.2. In Vitro Antimicrobial Effect of the Isolated Flavonoids

**Figure 2:**
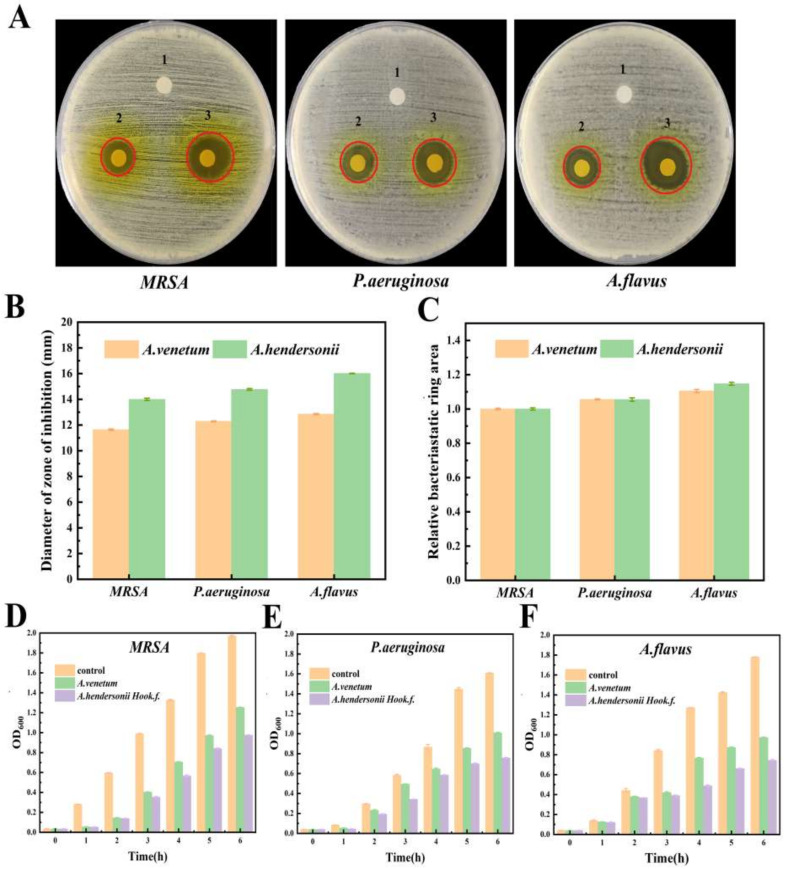
Digital images of inhibition zones of filter paper samples against: (**A**) Gram-positive bacteria MRSA; Gram-negative bacteria *P. aeruginosa*; Fungal *A. flavus* after treatment with different treatment groups (1) Cosolvent, (2) the FAV, (3) the FAH, respectively. The diameter of the filter paper was 6 cm. (**B**,**C**) Statistical column graphs of inhibition zones. The time-killing profiles of bacterial and fungal (**D**) MRSA, (**E**) *P. aeruginosa* and (**F**) *A. flavus* treated with serials of treatment, respectively. Plots were obtained from the measured optical density. Data are presented as mean ± SD, *n* = 3.

The antimicrobial activity of the isolated flavonoids of the *Apocynum* spp. were evaluated by the agar well diffusion method and time-dynamic microbicidal test. Figure 2A indicated that the flavonoid ingredients of both *A. venetum* (FAV) and *A. hendersonii* (FAH) exhibited significant antimicrobial activity to MRSA, *P. aeruginosa* and *A. flavus*. The diameters of inhibition zones for MRSA, *P. aeruginosa* and *A. flavus* with FAV extracts treatment were 11.6 mm, 12.3 mm and 12.8 mm, respectively and 14.0 mm, 14.7 mm and 16.0 mm, respectively, with FAH extracts treatment (Figure 2B). *A. hendorsonii* showed better performance for all the three microbes due to the highest zone of inhibition. In comparison to the three microbes, the isolated flavonoids were most active against the fungi *A. flavus*. Meanwhile, it showed higher activity against the Gram-negative *P. aeruginosa* compared to Gram-positive MRSA (Figure 2C). These different reactions may be ascribed to the differences of microbial membrane structure and component between the microbial strains [32,33]. The negative control group (co-solvent) had no inhibitory effect on the tested strains. Additionally, the long-term antimicrobial activity of the two kinds of flavonoid extracts against the three microbial strains conducted for 6 h is presented (Figure 2D–F). The results indicated that the isolated flavonoids from *A. hendersonii* leaf showed the strongest inhibitory effect and antibacterial activity, which was confirmed by the lowest optical density compared to that of *A. venetum*. As the main constituent of extracts of *A. hendersonii* leaf, the function of quercetin 3-sophoroside deserved further study.

### 3.3. Antibacterial Mechanism of the Isolated Active Flavonoid Ingredients

**Figure 3:**
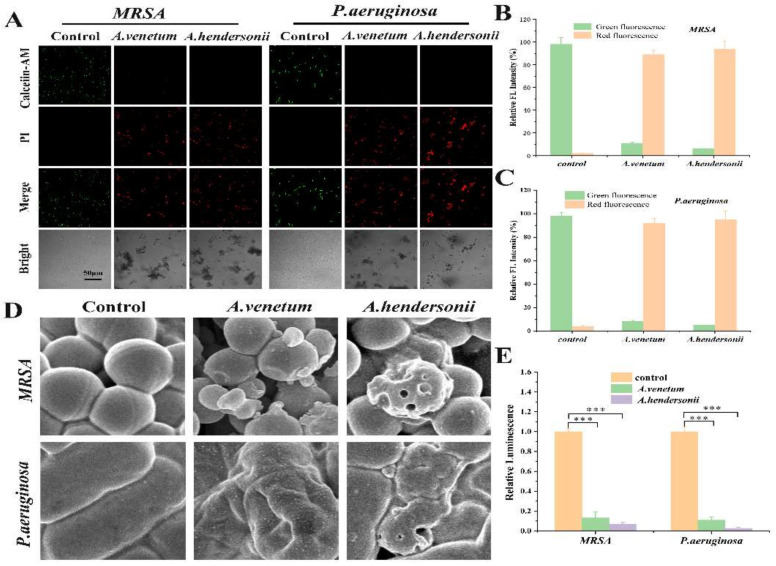
(**A**), Confocal fluorescent images of live and dead MRSA and *P. aeruginosa* cells after treatment with Cosolvent, the FAV and the FAH. Green fluorescence represents live bacteria stained with Calcein-AM, whereas red fluorescence represents dead bacteria stained with PI. (**B**,**C**,**E**), the corresponding quantitative assay of fluorescent intensity and the ATP level in MRSA and *P. aeruginosa* with different treatments. Inset data are ratios of live/dead bacteria. (**D**), SEM images of MRSA and *P. aeruginosa* treated with Cosolvent and the flavonoids extracts. Differences between groups with *** *p* < 0.001 was regarded as statistically significant.

The in vitro antibacterial activity of these isolated flavonoids were further evaluated by confocal images of live/dead staining assay (Figure 3A). As expected, all of the tested bacteria in the control group emitted strong green fluorescence, which means the negligible antimicrobial ability. The bacterial strains treated with the isolated flavonoids of both species simultaneously emitted green and red fluorescence, due to their bacterial-killing ability. Meanwhile, quantitative assay indicated that the strains treated with FAH exhibited stronger red fluorescence compared with FAV treated group (Figure 3B,C). As the green and red fluorescence intensities reflected the difference of bactericidal efficiency, the higher ratio of live/dead cells for both FAV treatments group in MRSA compared to *P. aeruginosa* indicated the better performance of the FAH. Besides, SEM images showed varying degrees of membrane damage of spherelike MRSA and rodshaped *P. aeruginosa* with FAV and FAH treatment. The cell membrane structure was more destructively broken with a hole in the MRSA and *P. aeruginosa* treated with FAH than FAV treated cells, compared with the control (Figure 3D).

ATP, as the most essential energy molecule, participates in many physiological processes of microorganism. Usually, ATP level will descend when the bacteria cells suffer from necrosis or apoptosis, the potential impairments in the mitochondria membrane [34,35]. By investigating the effect of FAV and FAH on ATP level, it was found that different treatments caused the decline of ATP level. The ATP level of MRSA and P. aeruginosa decreased about 84.6% and 93.9%, respectively, upon treatment with FAV. In contrast, FAH treatment caused ATP decrease about 94.8% and 97.3% for MRSA and *P. aeruginosa*, respectively (Figure 3E). The comparable ATP level demonstrated the good antibacterial activity of FAV and FAH.

### 3.4. Cytotoxicity and Hemolysis Ability of Flavonoid

**Figure 4:**
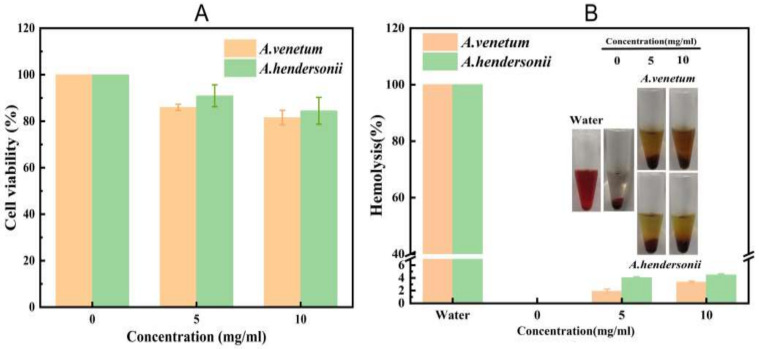
(**A**) Relative viabilities of NIH-3T3 cells after exposure to the isolated flavonoid ingredients of *A. venetum* and *A. hendersonii* of different concentrations. Error bars represent the standard error of three parallel experiments. (**B**) Hemolysis ratio of human blood incubated with different concentrations of these isolated flavonoid ingredients. The inset figure shows photographs of hemcytolysis.

**Figure 5:**
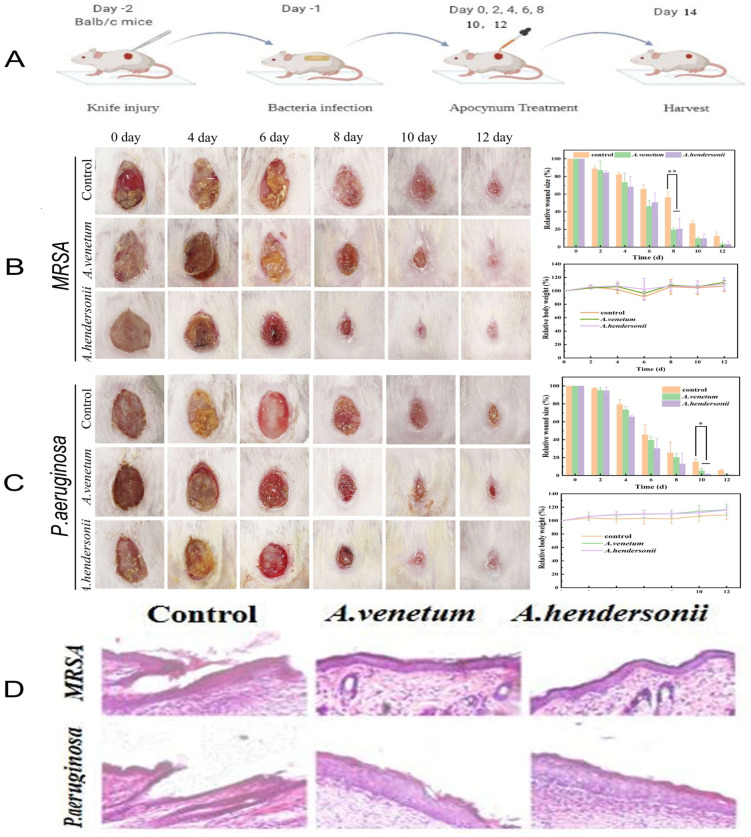
In vivo antibacterial activity of the FAV and FAH in Balb/c mice. (**A**), Schematic illustration for the construction of wound infection and treatment procedure. (**B**), Photographs of MRSA infected wounds, corresponding statistical graph of wound closure rates and changes of body weight of mice with different treatment at different time. (**C**), Photographs of *P. aeruginosa* infected wounds, corresponding statistical graph of wound closure rates and changes of body weight of mice with different treatment at different time. (**D**), H&E staining images of infected wound tissue after various treatments for 14 days. Differences between groups with * *p* < 0.05 and ** *p* < 0.01 were regarded as statistically significant.

The toxicity and hemolysis of the flavonoids were evaluated using standard MTT and hemolysis assay before starting the mice testing. The relative cell viability and hemolysis ability of the isolated flavonoids were assessed using mouse embryo cells (3T3 cells) and fresh red blood cells, respectively. As shown in Figure 4A, the viability of 3T3 cells still remains >80% even after incubating with high flavonoids concentrations (10 mg/mL) of the total flavonoids for 24 h, a selection of low cytotoxicity for both FAV and FAH. Meanwhile, good biocompatibility was observed as no significant erythrocyte damage was induced by the flavonoids (hemolysis rate was between 4.45% to 5% at a concentration of 10 mg/mL (Figure 5B). These results fully suggested the good biosafety of FAV and FAH for in vivo application.

### 3.5. In Vivo Microbial Infection Study

After demonstrating the in vitro antibacterial capability of FAV and FAH, we then investigated their efficiency on wound healing of female Balb/c mice infecting with MRSA and *P. aeruginosa* (Figure 5A). Samples were applied topically to assess the inhibition effect by the change of macroscopic appearance of the wounds compared with the control group. Figure 5B showed the representative photographs of MRSA-infected mice wound healing of MRSA-infected mice with various treatments at different time points. After 10 days, the area of MRSA-infected wounds in co-solvent, FAV and FAH groups decreased to 15.22%, 5.46% and 1.63%, respectively, indicating the accelerating effect of both FAV and FAH on wound healing with MRSA infection. Similar results were observed for *P. aeruginosa*-infected group mice (Figure 5C) as the lesion size of P. aeruginosa-infected wounds reduced to 9.69% and 9.75% in the FAV and FAH treated groups, respectively, compared to 26.71% for the control.

Meanwhile, the body weights of these treated mice were normal (Figure 5), reflecting the biological safety of the FAV and FAH. The corresponding hematoxylin and eosin (H&E) staining images of the tissues of the MRSA- or *P. aeruginosa*-infected mice wounds reflected the process of wound healing (Figure 5D). On the day 14 postoperation, apparent morphological features with new blood vessels and hair follicles was found in the groups treated with the FAH and FAV. In contrast, the other two control groups were badly damaged. Meanwhile, thicker granulation tissue was observed in the *P. aeruginosa* infected mice compared with the MRSA infected group. In addition, the treatment of the FAH against MRSA and *P. aeruginosa* bacteria groups showed complete and smooth epidermal tissue, comparing with slight damage in FAV treated mice. These results showed the comparable effect between FAH and FAV on wound healing. Meanwhile, it can be concluded that the total flavonoids from the two species possess high healing efficiency on wound healing.

### 3.6. Biological Safety Investigation

**Figure 6:**
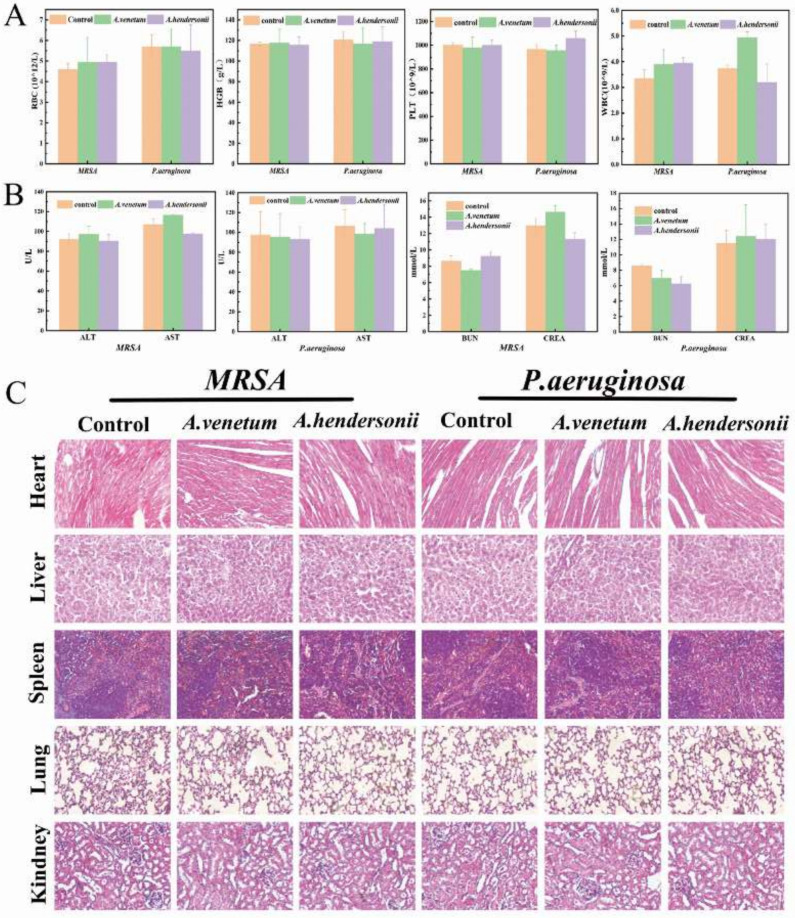
In vivo biosafety of the active flavonoid ingredients isolated from the two *Apocynum* spp. (**A**), Blood routine assay of red blood cell (RBC), hemoglobin (HGB), Platelet (PLT) and white blood cells (WBC). (**B**), Histopathological examination of major organs including the heart, liver, spleen, lungs, and kidneys at day 14 after different treatments. (**C**), H&E staining of the heart, liver, spleen, lung, and kidney at day 12 after with different treatments.

Considering the practical and safety use of antibacterial agents in vivo, it is essential to carry out the biocompatibility assay of blood and the main organs. As revealed in Figure 6A,B, the common blood biochemical parameters (RBC, HGB, PLT and WBC) and the liver (AST and ALT) and Kidney markers (BUN and CREA) of mice with bacterial infections showed no significant changes compared with the normal mice. In addition, H&E staining (Figure 6C) results further indicate normal morphological features without obvious abnormalities and inflammatory lesions to all investigated organs. These results clearly indicated the good biocompatibility and ultra-low toxicity of the FAV and FAH in vivo.

## 4. Conclusions

In summary, the accurate and reliable UPLC-ESI-MS/MS method was successfully used for the characterization of the active flavonoid ingredients from *A. venetum* (FAV) and *A. hendersonii* (FAH). The results indicated the high content of hyperoside and isoquercet in FAV, but quercetin-3-*O*-β-D-glucosyl-β-D-glucopyranoside in FAH based on this method. Furthermore, both of them displayed high antioxidant activity and inhibiting effect on MDR gram-positive bacteria (MRSA), gram-negative bacteria (*P. aeruginosa*) and fungal (*A. flavus)* in vitro and in vivo with high biocompatibility. Mechanism exploration indicated that these active flavonoids can disrupt cell membrane integrity, inhibit ATP synthesis and damage metabolism to achieve antimicrobial activity. FAV and FAH decreased the possibility of persistent bacterial infection and suppuration and promoted wound healing in vivo. Based on the above results, we envision that the UPLC-ESI-MS/MS method showed timesaving and sensitive advantages for characterization and accurate quantitation of active flavonoid ingredients with antimicrobial and antioxidant effect from *Apocynum* spp., which hold potential applications for diagnosis and therapy of bacterial infections.

## Figures and Tables

**Table 1:** Yielding and the antioxidant potency of the isolated flavonoids.

	Content (mg/g)	IC50_(ABST+)_ (ug/mL)	IC_50(DPPH)_ (ug/mL)	IC_50(Bleaching)_ (ug/mL)
Flavonoids isolated from *A. venetum*	15.35 ± 0.47	201.21 ± 12.44	35.68 ± 2.53	31.02 ± 1.54
Flavnonoids isolated from *A. hendersonii*	13.28 ± 0.22	195.47 ± 10.25	33.52 ± 3.12	24.15 ± 1.67
Rutin (positive control)	/	69.14 ± 2.22	7.23 ± 0.34	17.59 ± 0.16

IC50 values are presented with their respective 95% confidence limits.

## Data Availability

Data is contained within the article and Supplementary Materials.

## Supplementary Materials

The following are available online at https://www.mdpi.com/article/10.3390/antiox10121901/s1, Figure S1: UPLC-ESI-MS/MS data of flavonoid ingredients characterized in the leaf extracts of *A. venetum* and *A. hendersonii*.
