# Supplementary material for: UPLC-ESI-MS/MS Based Characterization of Active Flavonoids from Apocynum spp. and Anti-Bacteria Assay
Source: Antioxidants (Basel). 2021 Nov 27;10(12):1901. doi: 10.3390/antiox10121901 (PMC8750526; doi:10.3390/antiox10121901)
Supplement: Supplementary file 1 [file antioxidants-10-01901-s001.zip › antioxidants-1440101-supplementary.pdf]

Suplementary materials.1. UPLC-ESI-MS/MS data of flavonoid ingredients characterized in the leaf extracts of *A. venetum* and *A. hendersonii.*

	Peak	RT (min)	UV λmax	MS [m/Z]-	Formula	Tentative identification
A．venetum	1	10.113	202，256，355	464	$C_{21}H_{20}O_{12}$	Hyperoside
	2	10.320	203，256，354	464	$C_{21}H_{20}O_{12}$	Isoquercetin
	3	11.277	203，256，355	550	$C_{24}H_{22}O_{15}$	Quercetin-3-O-(6-O-malonyl)- Galactoside
	4	11.420	203，256，355	550	$C_{24}H_{22}O_{15}$	Quercetin-3-O-(6-O-malonyl)-glucoside
	5	12.693	203，256，355	505	$C_{23}H_{22}O_{13}$	Quercetin-3-O-(6-O-acetyl)-galactoside
A．hendersonii	1	7.433	203，256，354	626	$C_{27}H_{30}O_{17}$	Quercetin 3-sophoroside (Baimaside)
	2	10.450	203，256，354	464	$C_{21}H_{20}O_{12}$	Isoquercetin
	3	11.383	203，256，355	549	$C_{24}H_{22}O_{15}$	Quercetin-3-O-(6-O-malonyl)-Galactoside
